# Interaction between depressive symptoms and social support on frailty risk among patients with chronic heart failure

**DOI:** 10.3389/fcvm.2026.1761191

**Published:** 2026-06-25

**Authors:** Mingjuan Guo, Yaohong Pan, Tingting Liao, Ruping Meng

**Affiliations:** 1 School of Nursing, Guangxi Health Science College, Nanning, China; 2 Department of Nursing, The Third People's Hospital of Nanning, Nanning, China; 3 Department of Cardiovascular Medicine, The First Affiliated Hospital of Guangxi Medical University, Nanning, China

**Keywords:** chronic heart failure, depressive symptoms, frailty, interaction effect, social support

## Abstract

**Background:**

Frailty is highly prevalent among patients with chronic heart failure (CHF). Depressive symptoms and social support are significant psychosocial factors associated with frailty, yet how they interact to jointly relate to frailty remains insufficiently explored.

**Objective:**

This study aimed to investigate the interaction effect between depressive symptoms and social support on the presence of frailty in patients with CHF, to inform the development of targeted intervention strategies.

**Methods:**

A cross-sectional study was conducted among 380 patients with CHF from a tertiary hospital in Nanning, China. Data were collected using a general information questionnaire, the Patient Health Questionnaire-9 (PHQ-9), the Social Support Rating Scale (SSRS), and the FRAIL Scale. Logistic regression and interaction analyses were performed to evaluate correlations and interactions.

**Results:**

The prevalence of frailty was 41.3%. Logistic regression analysis identified depressive symptoms (odds ratio [OR] = 7.37, 95% confidence interval [CI]: 4.29–12.63) and low social support (OR = 5.88, 95% CI: 3.57–10.00) as associated factors for frailty (both *P* < 0.01). A significant multiplicative interaction was observed (OR = 0.23, 95% CI: 0.08–0.66, *P* = 0.007). Additive interaction analysis further confirmed a synergistic effect (relative excess risk due to interaction [RERI] = 19.19, 95% CI: 9.64–46.94; attributable proportion [AP] = 0.84, 95% CI: 0.72–0.92; synergy index [S] = 8.29, 95% CI: 4.19–37.19).

**Conclusion:**

Depressive symptoms and social support were significantly associated with frailty, and their interaction demonstrated a synergistic pattern. Longitudinal studies are warranted to explore the temporal nature of these associations. However, the single-center, regional, and male-predominant nature of the sample limits generalizability.

## Background

Chronic heart failure (CHF) is a complex clinical syndrome and a major global public health challenge, with prevalence projected to rise by 46% by 2030 alongside substantial economic burden (1–3). In China, despite a relatively low national prevalence, the combination of a large patient population, rapid demographic aging, and high rates of comorbidities such as hypertension and diabetes renders CHF a serious public health concern (1). Current clinical management of CHF primarily centers on optimizing pharmacologic therapy and improving physiological parameters. However, a growing body of evidence indicates that patient health outcomes are not solely determined by biological factors but are also profoundly modulated by an integrated network of psychological and social determinants. Frailty as a clinical syndrome is particularly common in patients with CHF (4). An extensive meta-analysis (5) encompassing 26 studies and 6,896 CHF patients revealed a pooled frailty prevalence of 44.5%, indicating that it affects nearly half of all heart failure patients. In patients with CHF, energy depletion and chronic inflammation are linked to catabolic state abnormalities, which in turn correlate with heightened inflammation and metabolic dysregulation, coinciding with aggravating myocardial injury and worsening heart failure symptoms (6, 7). Compared with non-frail counterparts, CHF patients with frailty show a significantly higher symptom burden, characterized by twice the dyspnea, a 75% greater severity of sleep disorder and depressive symptoms, and markedly reduced quality of life (8). The functional dependence and reduced self-care capacity observed with frailty significantly correspond to increase reliance on external support from both families and healthcare systems thereby highlighting the essential protective role of social support as a modifiable resource in disease management.

Previous studies (9) have confirmed that psychological and social factors are closely related to the presence of frailty in patients with CHF. The estimated overall prevalence of depressive symptoms in patients with CHF is 20%–30% (10). Dysregulated inflammatory pathways represent a key mechanistic link between depressive symptoms and CHF, with elevated inflammatory biomarkers correlating with both myocardial remodeling and the pathogenesis of depression (11, 12). Depressive symptoms are accompanied by reduced physical activity, low mood, and enhanced stress responses, which together show a relationship with a higher frailty status and greater cardiovascular risk (13). A cross-sectional study conducted by Jung et al. revealed that among 382 participants, those with depressive symptoms exhibited a higher likelihood of frailty compared to those without depressive symptoms (OR = 5.25, 95% CI: 2.55–10.83) (14). A meta-analysis (15) demonstrated that heart failure patients with comorbid depression had a two-fold higher risk of mortality and cardiovascular events (hazard ratio[HR] = 1.57, 95% CI: 1.30–1.89). Furthermore, depressive symptoms coincide with greater physical discomfort and fatigue, which are strongly correlated with impaired self-management abilities and social functioning, thereby relating to an overall decline in quality of life (16). Depressive symptoms, functional decline, and frailty coexist and interact as core determinants that are closely linked to increased clinical risk in CHF patients. These findings collectively underscore that depressive symptoms are not merely a psychological comorbidity but a pivotal modifiable factor for frailty in CHF patients, warranting routine screening and integrated psychosocial interventions in clinical practice.

Social support is defined as the overall level of material and emotional resources an individual obtains from their environment, comprising both positive social interactions and perceived external assistance (17). As a critical external resource, social support demonstrates a protective relationship in patients with CHF. High social support corresponds to reduced psychological stress and better physiological outcomes, as reflected by lower cardiovascular reactivity (18). Low social support is linked to a higher likelihood of frailty, whereas sustained support shows a consistent protective pattern against its presence (19). Depressive symptoms often coexist with and are interrelated with frailty, which in turn restricts a patient's social capacity, thus correlating with diminished social support and forming a reciprocal cycle (20). Frailty has been connected to chronic inflammation mediated by inflammatory factors, while stronger social support has been observed in conjunction with lower levels of pro-inflammatory cytokines (21). Strong social support is related to a lower likelihood of frailty through mechanisms such as providing emotional comfort, assisting with disease management, and buffering stress responses (22).

Collectively, depressive symptoms, social support and frailty do not exist in isolation but form an interconnected and dynamically evolving biopsychosocial cycle. The physical functional decline observed with frailty progressively limits social activities and diminishes social support networks, thereby contributing to a higher likelihood of depressive symptoms (23). Depressive symptoms are correlated with reduced treatment adherence and heightened inflammatory responses, which together show a relationship with frailty status (24). Furthermore, the associated negative affect and social withdrawal may further interact with social support, thereby reinforcing this detrimental pattern (13). Inadequate social support not only amplifies psychological distress and susceptibility to depression but also is linked to reduced resilience to disease stress, thereby coinciding with a higher frailty status (25). However, existing studies have largely examined depressive symptoms and social support as independent risk or protective factors, with few investigations exploring their interaction in the development of frailty. In patients with CHF who experience both physical and psychological stress, how depressive symptoms and social support jointly relate to frailty risk and whether an interactive effect exists remain poorly understood.

Therefore, this study is grounded in the biopsychosocial model, which posits that health outcomes are shaped by the dynamic interplay of biological, psychological, and social factors (26). In patients with CHF, biological factors such as chronic inflammation and energy depletion create a physiological vulnerability to frailty. Psychological factors particularly depressive symptoms, are correlated with frailty status through behavioral and neuroendocrine pathways. Social factors, notably social support, may show a protective connection by buffering stress and promoting adaptive coping. Guided by this integrative theoretical framework, we hypothesize that social support moderates the relationship between depressive symptoms and frailty risk.

To test this hypothesis, the present study investigates the interaction between depressive symptoms and social support on frailty risk among Chinese patients with CHF. The findings are expected to provide evidence for identifying high-risk individuals and developing integrated intervention strategies that combine psychological therapy with family-based support.

## Methods

### Study design and participants

A cross-sectional study was conducted among 380 patients with CHF recruited from the Department of Cardiovascular Medicine of a tertiary hospital in Nanning, Guangxi, China, between September 2024 and June 2025. Potential participants were identified by screening the hospital information system for CHF diagnoses using a convenience sampling method. Eligible patients were approached approximately 24 h after admission, once their condition had stabilized and they were not undergoing active treatment. After obtaining written informed consent, a trained researchers administered the survey using paper-based questionnaires. Participants completed the questionnaires independently after receiving instructions. The researchers reviewed each questionnaire on the spot to identify and address any missing or ambiguous responses. Clinical data were extracted from the hospital information system. The inclusion criteria were as follows: (1) Diagnosis of CHF according to the “Chinese Guidelines for the Diagnosis and Treatment of Heart Failure 2024” (27); (2) Age≥18 years; (3) Willingness to participate in the study. The exclusion criteria were as follows: (1) Diagnosis of dementia or other major neurocognitive disorders; (2) Use of psychotropic or antiepileptic medications; (3) Presence of severe neurological conditions (such as Parkinson's disease, stroke with significant functional impairment, or other neurodegenerative disorders); (4) Any condition that would preclude cooperation with study protocols. To ensure data quality, all data were double-entered by two independent researchers for cross-checking. This study has been reviewed and approved by the Ethics Committee of the First Affiliated Hospital of Guangxi Medical University (Number: 2024-K423-01). All participants voluntarily joined and signed informed consent forms. The collected data and information were encoded and treated confidentially.

### Sample size estimation

The sample size was calculated based on a reported a prevalence of frailty of 68.75% among patients with CHF (28)， With a significance level (α) of 0.05 and a margin of error (δ) of 0.05, the minimum required sample size was 330. To account for potential invalid questionnaires or data loss, the sample size was increased by 10%, resulting in 363 participants. Ultimately, 380 patients with CHF were enrolled in this study.

$$N=\frac{Z_{\alpha /2}^{{}^{2}}\cdot \pi \cdot (1-\pi )}{\delta^{2}}$$

### Measurement

#### General information questionnaire

It includes the following contents: (1)General demographic information of the patient: age, sex, body mass index (BMI), educational level, marital status; (2)Disease-related data: smoking history, alcohol history, duration of illness, New York Heart Association (NYHA) class, history of hypertension, history of diabetes, history of stroke, history of coronary heart disease, history of atrial fibrillation; (3)Examination report and laboratory indicators: hemoglobin, high-sensitivity C-reactive protein(hs-CRP), total cholesterol, triglycerides, serum creatinine, aspartate aminotransferase, alanine aminotransferase, N-terminal pro-B-typenatriuretic peptide (NT-ProBNP), left ventricular ejection fraction (LVEF).

#### Patient health questionnaire-9 depression scale (PHQ-9)

The scale was developed by American scholars Spitzer et al. (29) and translated into Chinese by Bian et al. (30). It is an important tool for screening and assessing depressive symptoms, consisting of 9 items to evaluate the feelings of the subjects in the past two weeks. The total score ranges from 0 to 27 points. For the purpose of this study, the total score was dichotomized into “no depressive symptoms” (0–4) and “depressive symptoms” (≥5), indicating the presence of clinically relevant depressive symptoms rather than a clinical diagnosis of depression. The Cronbach'sα coefficient of this scale is 0.898 in this study.

#### Social support rating scale (SSRS)

The Social Support Rating Scale (SSRS), a validated questionnaire made by Xiao et al. (31), was used to measure social support. The scale, which includes 10 items in total, contains three parts: objective support (three items), subjective support (four items) and utilization of support (three items). The total score ranges from 0 to 66, with a higher score indicating a greater level of perceived social support. Since the SSRS lacks a universally accepted clinical cut-off, we dichotomized social support using the median score of the study sample as the threshold, which also approximated 50% of the maximum possible score (0–66). Accordingly, participants scoring ≥37 were classified as having high social support, while those scoring <37 were classified as having low social support. The SSRS demonstrated good internal consistency, with a Cronbach'sα coefficient of 0.801 in this study.

#### The FRAIL (fatigue, resistance, ambulation, illness, loss of weight) scale

The FRAIL Scale developed by the International Academy on Nutrition and Aging (IANA) and the International Association of Gerontology and Geriatrics (IAGG) (32). It has been proven to be effective for measuring physical frailty in older Chinese CHF patients (33). It is a brief but valid instrument with five physiological indicators for effectively identifying frailty or per-frailty status. The total score ranges from 0 to 5, with scores of 0–2 indicating “no frailty” and scores of 3–5 indicating “frailty”. The Cronbach's*α* coefficient of this scale is 0.847 in this study.

### Statistical analysis

Normality of continuous variables was assessed using the Shapiro–Wilk test. Normally distributed continuous data are presented as the mean ± standard deviation and between-group comparisons were performed using the t-test after confirming homogeneity of variances with Levene's test. Non-normally distributed continuous data are expressed as the median with interquartile range (P25, P75), and the Wilcoxon rank-sum test was used for comparisons. Categorical data are summarized as frequency (percentage), and group differences were assessed using the chi-square test.

Multivariable logistic regression was employed to examine the associations of depressive symptoms and social support with frailty in patients with CHF. Multicollinearity among independent variables was assessed using the variance inflation factor (VIF), with VIF < 5 indicating no significant collinearity. Variables included in the multivariable model were selected based on statistical significance in univariate analyses (*P* < 0.05). All hypothesis tests were two-sided, with a significance level of α=0.05. The multivariable models were adjusted for age, marital status, NYHA class, hypertension, coronary heart disease, and log-transformed hs-CRP to account for potential confounding by systemic inflammation.

Additive and multiplicative interaction analyses were conducted using logistic models in R software (version 4.2.3). Multiplicative interaction was assessed by the odds ratio (OR) and its 95% confidence interval (CI) for the product term of the two indicators; if the 95% CI included 1, no multiplicative interaction was considered present. Additive interaction was evaluated using the relative excess risk due to interaction (RERI), the attributable proportion (AP), and the synergy index (S), calculated according to the algorithms described by Knol and VanderWeele (34). The 95% CIs for RERI, AP, and S were obtained using the bootstrap method (500 replications). Absence of additive interaction was defined when the 95% CI for RERI and AP included 0, and the 95% CI for S included 1. Stratified analyses were additionally performed to examine the association between depressive symptoms and frailty separately in low and high social support groups, adjusting for the same covariates as in the main model.

## Results

### Baseline characteristics

The study enrolled 380 patients diagnosed with CHF. Demographic data revealed a mean age of 61.33 ± 12.99 years, with 268 (70.53%) male and 112 (29.47%) female participants. Specifically, the prevalence rates among these patients were 41.3% for frailty and 60.79% for depressive symptoms. Normality of continuous variables was assessed using the Shapiro–Wilk test, and homogeneity of variances was evaluated using Levene's test. Age was found to be normally distributed in both groups (*P* > 0.05), and equal variances were assumed (*P* > 0.05). Statistically significant differences were observed in NYHA class, hypertension, coronary heart disease, social support, and depressive symptoms among CHF patients with frailty (*P* < 0.05). In contrast, no significant differences were found in terms of gender, age, BMI, education level, smoking history, or alcohol consumption history (*P* > 0.05) (Table 1).

**Table 1:** Baseline characteristics and differences in frailty (*n* = 380).

Variables	Total (*n* = 380)	No frailty (*n* = 223)	Frailty (*n* = 157)	*Statistic*	*P-value*
Age, Mean ± SD	61.33 ± 12.99	60.30 ± 13.19	62.79 ± 12.60	−1.845	0.066
Sex, *n* (%)				0.933	0.334
Male	268 (70.53%)	162 (72.65%)	106 (67.52%)		
Female	112 (29.47%)	61 (27.35%)	51 (32.48%)		
BMI, Mean ± SD	24.21 ± 4.18	24.40 ± 4.24	23.94 ± 4.10	1.055	0.292
Educational level, *n* (%)				5.667	0.129
Primary school or below	115 (30.26%)	77 (34.53%)	38 (24.20%)		
Junior high school	145 (38.16%)	80 (35.87%)	65 (41.40%)		
High school or technical secondary school	66 (17.37%)	39 (17.49%)	27 (17.20%)		
College or above	54 (14.21%)	27 (12.11%)	27 (17.20%)		
Marital status, *n* (%)				3.189	0.074
Unmarried	49 (12.89%)	35 (15.70%)	14 (8.92%)		
Married	331 (87.11%)	188 (84.30%)	143 (91.08%)		
Smoking history, *n* (%)				0.262	0.609
No	289 (76.05%)	165 (73.99%)	122 (77.71%)		
Yes	91 (23.95%)	56 (25.01%)	35 (22.29%)		
Alcohol history, *n* (%)				0.001	0.982
No	281 (73.95%)	165 (73.99%)	116 (73.89%)		
Yes	99 (26.05%)	58 (26.01%)	41 (26.11%)		
Duration of illness, *n* (%)				0.360	0.835
＜3	259 (68.16%)	152 (68.16%)	107 (68.15%)		
3–5	40 (10.53%)	25 (11.21%)	15 (9.55%)		
＞5	81 (21.32%)	46 (20.63%)	35 (22.29%)		
NYHA class, *n* (%)				11.775	**0.003**
Class Ⅱ	157 (41.32%)	103 (46.19%)	54 (34.39%)		
Class Ⅲ	149 (39.21%)	89 (39.91%)	60 (38.22%)		
Class Ⅳ	74 (19.47%)	31 (13.90%)	43 (27.39%)		
History of hypertension, *n* (%)				8.027	**0.005**
No	167 (43.95%)	112 (50.22%)	55 (35.03%)		
Yes	213 (56.05%)	111 (49.78%)	102 (64.97%)		
History of diabetes, *n* (%)				0.034	0.854
No	274 (72.11%)	160 (71.75%)	114 (72.61%)		
Yes	106 (27.89%)	63 (28.25%)	43 (27.39%)		
History of stroke, *n* (%)				0.012	0.914
No	321 (84.47%)	188 (84.30%)	133 (84.71%)		
Yes	59 (15.53%)	35 (15.70%)	24 (15.29%)		
History of coronary heart disease, *n* (%)				4.625	**0.032**
No	134 (35.26%)	89 (39.91%)	45 (28.66%)		
Yes	246 (64.74%)	134 (60.09%)	112 (71.34%)		
History of atrial fibrillation, *n* (%)				0.782	0.376
NO	286 (75.26%)	172 (77.13%)	114 (72.61%)		
Yes	94 (24.74%)	51 (22.87%)	43 (27.39%)		
LVEF, Mean ± SD	0.51 ± 0.16	0.51 ± 0.16	0.50 ± 0.17	0.575	0.566
Hemoglobin, Mean ± SD (g/L)	129.57 ± 25.12	129.17 ± 24.32	130.15 ± 26.29	−0.377	0.707
Hs-CRP, Median (IQR) (mg/L)	3.11 (0.80, 10.00)	2.40 (0.80, 10.00)	3.68 (0.80, 10.00)	−0.564	0.562
Total cholesterol, Median (IQR) (mmol/L)	3.92 (3.24, 4.83)	3.83 (3.20, 4.81)	4.08 (3.28, 4.86)	−1.192	0.233
Triglyceride, Median (IQR) (mmol/L)	1.20 (0.92, 1.78)	1.19 (0.93, 1.78)	1.21 (0.90, 1.75)	0.275	0.784
Serum creatinine, Median (IQR) (µmol/L)	94.00 (74.00, 123.25)	94.00 (74.00, 123.50)	93.00 (74.00, 123.00)	0.364	0.716
Alanine Aminotransferase, Median (IQR) (U/L)	21.00 (14.00, 34.00)	21.90 (14.00, 32.00)	20.00 (13.00, 37.00)	0.460	0.645
Aspartate Aminotransferase, Median (IQR) (U/L)	23.00 (18.00, 33.00)	24.00 (18.00, 33.00)	23.00 (19.00, 33.00)	−0.203	0.839
NT-proBNP (pg/mL)	2,080.50 (565.50, 5,413.75)	1,939.00 (614.50, 5,351.50)	2,409.00 (564.00, 5,772.00)	−0.751	0.453
Social support, *n* (%)				46.160	**< 0.001**
Low	207 (54.47%)	89 (39.91%)	118 (75.16%)		
High	173 (45.53%)	134 (60.09%)	39 (24.84%)		
Depressive symptoms, *n* (%)				54.389	**< 0.001**
No	149 (39.21%)	122 (54.71%)	27 (17.20%)		
Yes	231 (60.79%)	101 (45.29%)	130 (82.80%)		

Bold values indicates that *P-values* are < 0.05.

### Multiplicative interaction between depressive symptoms and social support

After adjusting for confounders including age, marital status, NYHA class, hypertension, and coronary heart disease, and log-transformed hs-CRP, multivariate logistic regression analysis revealed that CHF patients with depressive symptoms had a significantly higher risk of frailty compared to those without (*P* < 0.05). Furthermore, low social support was associated with an increased risk of frailty relative to high social support (*P* < 0.05). A statistically significant multiplicative interaction was identified between depressive symptoms and social support (*P* < 0.05) (Table 2). Social support exerted a significant negative moderating effect on the relationship between depressive symptoms and frailty. Specifically, a high level of social support substantially attenuated the promoting effect of depressive symptoms on frailty risk. Log-hs-CRP itself was not independently associated with frailty (OR = 0.98, 95% CI: 0.84–1.15, *P* = 0.82).

**Table 2:** Multiplicative interaction between depressive symptoms and social support on frailty risk.

Variable	Model 1		Model 2	
	OR (95% CI)	*P*-value	OR (95% CI)	*P*-value
Depressive symptoms	7.37 (4.29–12.63)	<0.001	7.23 (4.13–12.65)	<0.001
Social support	0.17 (0.10–0.28)	<0.001	0.17 (0.10–0.29)	<0.001
Depressive symptoms×Social support	0.23 (0.08–0.66)	0.007	0.23 (0.08–0.69)	0.009

Model 1 had no controlled variables; Model 2 controlled for age, marital status, NYHA class, hypertension, coronary heart disease, and log-transformed hs-CRP.

### Stratified analysis by social support level

To further characterize the interaction, we examined the association between depressive symptoms and frailty separately among patients with low vs. high social support, adjusting for adjusting for age, marital status, NYHA class, hypertension, coronary heart disease, and log- hs-CRP (the same covariates used in the main model). In the low social support group (*n* = 207), depressive symptoms were significantly correlated with frailty (OR = 12.97, 95% CI: 6.33–26.55, *P* < 0.001). In the high social support group (*n* = 173), the magnitude of this correlation was substantially weaker (OR = 2.74, 95% CI: 1.18–6.39, *P* = 0.019). These stratified results support a modifying effect of social support on the relationship between depressive symptoms and frailty.

### Additive interaction between depressive symptoms and social support

Univariate logistic regression analysis indicated that, compared to patients without depressive symptoms and with high social support, those with both depressive symptoms and low social support had a 22.83-fold increased risk of frailty (95% CI: 10.26–50.80). After adjusting for confounding factors, the results of multivariate logistic regression analysis showed that compared with the population without depressive symptoms and with a high level of social support, both having depressive symptoms alone and having a low level of social support alone increased the risk of frailty (OR =2.65 and 2.00, respectively). Patients with CHF who had both depressive symptoms and low social support had a 22.36-fold higher risk of frailty compared to those without depressive symptoms and with high social support (95% CI: 9.88–50.61). A significant additive interaction was observed between depressive symptoms and low social support concerning the prevalence of frailty in patients with CHF. Analyses using measures of additive interaction further confirmed a substantial synergistic effect. The estimated indices were as follows: the RERI was 19.19 (95% CI: 9.64–46.94), the AP was 0.84 (95% CI: 0.72–0.92), and the S was 8.29 (95% CI: 4.19–37.19).

The additive interaction between depressive symptoms and social support continued to demonstrate statistical significance even after adjusting for age, marital status, NYHA class, hypertension, and coronary heart disease and log- hs-CRP. The RERI value was 18.45 (95% CI: 8.80–52.99), which indicates that the excess relative risk of frailty attributable to the interaction between depressive symptoms and low social support was 18.45. The AP was 0.84 (95% CI: 0.70–0.93), meaning that approximately 84% of frailty cases among CHF patients with both exposures were attributable to the interaction. The S was 7.99 (95% CI: 3.90–40.42), suggesting that the risk of frailty when both depressive symptoms and low social support are present is 7.99 times the sum of the risks associated with each factor alone (Table 3).

**Table 3:** Additive interaction between depressive symptoms and social support on frailty risk.

Depressive symptoms	Social support	Total (*n*=380)	Frailty (*n*=157)	Prevalence of frailty, *n* (%)	Model 1		Model 2	
					OR (95% CI)	*P* value	OR (95% CI)	*P* value
No	High level	70	9	12.85%	Reference		Reference	
No	Low level	79	18	22.78%	1.95 (0.83–4.59)	0.128	2.00 (0.84–4.73)	0.114
Yes	Low level	128	100	78.15%	22.83 (10.26–50.80)	<0.001	22.09 (9.79–49.87)	<0.001
Yes	High level	103	30	29.13%	2.69 (1.20–5.99)	0.016	2.65 (1.17–5.96)	0.019
Interaction effect								
RERI					19.19 (9.64–46.94)	<0.001	18.45 (8.80–52.99)	<0.001
AP					0.84 (0.72–0.92)	<0.001	0.84 (0.70–0.93)	<0.001
SI					8.29 (4.19–37.19)	<0.001	7.99 (3.90–40.42)	0.020

Model 1 had no controlled variables; Model 2 controlled for age, marital status, NYHA class, hypertension, coronary heart disease, and log-transformed hs-CRP.

### Subgroup analyses

Subgroup analyses were performed to examine whether the multiplicative interaction between depressive symptoms and social support varied across different patient subgroups, including age, sex, NYHA class, LVEF, and hypertension (Table 4). The interaction remained statistically significant in patients older than 63 years (OR = 0.18, 95% CI: 0.03–0.94, *P* = 0.042), in male patients (OR = 0.14, 95% CI: 0.03–0.60, *P* = 0.008), in those with NYHA class Ⅲ (OR = 0.08, 95% CI: 0.01–0.50, *P* = 0.007), and in both LVEF subgroups (LVEF < 50%: OR = 0.12, 95% CI: 0.02–0.76, *P* = 0.024; LVEF ≥ 50%: OR = 0.17, 95% CI: 0.03–0.80, *P* = 0.026). No significant interaction was observed in younger patients, female patients, or patients (all *P* > 0.05). In hypertensive and non-hypertensive patients, the interaction was not statistically significant (both *P* > 0.05), although the direction of effect was consistent.

**Table 4:** Subgroup analysis for the multiplicative interaction between depressive symptoms and social support on frailty risk.

Subgroup	Level	N	OR for interaction (95% CI)	*P* value
Age	≤63 years	194	0.30 (0.07–1.41)	0.129
	>63 years	186	0.18 (0.03–0.94)	0.042
Sex	Male	268	0.14 (0.03–0.60)	0.008
	Female	112	0.66 (0.11–4.15)	0.660
NYHA class	Ⅱ	157	0.47 (0.09–2.38)	0.362
	Ⅲ	149	0.08 (0.01–0.50)	0.007
	Ⅳ	74	0.89 (0.04–151.60)	0.949
LVEF	＜50%	185	0.12（0.02–0.76）	0.024
	≥50%	195	0.17（0.03–0.80）	0.026
Hypertension	Yes	213	0.28 (0.07–1.08)	0.066
	No	167	0.22 (0.03–1.85)	0.162

## Discussion

This study found that the prevalence of frailty among patients with CHF was 41.3%. This prevalence is lower than the 70.6% reported by Yang et al. (35) among 323 hospitalized CHF patients from three tertiary hospitals in Wuhan. Yang et al. employed the Tilburg Frailty Indicator (TFI), a multidimensional instrument encompassing physical, psychological, and social domains, whereas the present study utilized the FRAIL scale, which focuses exclusively on physical frailty. Furthermore, their study population comprised a higher proportion of patients with advanced heart failure (NYHA class III–IV) and lower educational attainment, both of which are well-established correlates of higher frailty prevalence. Additionally, a cross-sectional study by Tang et al. (28) involving 256 hospitalized CHF patients in China reported a frailty prevalence of 68.75%. Their findings identified age, nutritional status, and NT-proBNP level as factors linked to frailty, while albumin and LVEF showed a relationship with lower frailty prevalence. A reciprocal pattern exists between CHF and frailty. Physical deconditioning systemic inflammation, neuroendocrine activation, and malnutrition are considered key pathways through which heart failure is connected to sarcopenia, which in turn coincides with frailty (36). Moreover, the decline in multisystem physiological reserve observed with frailty corresponds to reduced capacity to compensate for acute stress, poor treatment tolerance, diminished self-management ability, and a greater burden of comorbidities (37). These factors collectively show a connection to significantly worsened clinical prognosis and quality of life in heart failure patients. This underscores the importance of integrating frailty assessment into the routine management of heart failure. Future treatment strategies should shift toward a comprehensive model that combines rehabilitation, nutritional support, and multi-morbidity care to address this interrelated condition.

Frailty arises from multiple contributing factors, among which depressive symptoms are a demonstrated independent factor (38). A cross-sectional study conducted in Korea (39) demonstrated that CHF patients with depressive symptoms had a significantly higher risk of frailty compared to those without depressive symptoms (OR = 4.789，*P* < 0.001). The link between depressive symptoms and frailty may be related to reduced social engagement and physical mobility, as well as increased sedentary time, fall risk, weight loss, and malnutrition, thereby coinciding with a higher frailty status (40). Conversely, frailty itself may show a connection to the presence of depressive symptoms. A large-scale prospective study utilizing the UK Biobank (41) revealed that frailty is correlated with systemic low-grade inflammation, characterized by rises in C-reactive protein, neutrophils, and white blood cells. This may be linked to the occurrence of depressive symptoms by activating the immune inflammatory pathway and the process of adrenocorticotropic hormone secretion. These findings suggest that clinicians should integrate validated screening tools for the early detection of depressive symptoms into the routine management of CHF. For patients with high frailty status who also exhibit depressive symptoms, cardiac rehabilitation plans should incorporate psychosocial support and behavioral activation strategies. Encouraging safe social participation and setting attainable activity goals can improve physical function and mood, which collectively may be correlated with reduced frailty risk.

Furthermore, multiple studie (42, 43) have indicated that higher levels of social support are linked to a lower frailty risk in patients with CHF. A cross-sectional study of community-dwelling older adults in Thailand (44) identified social support as a factor correlated with health-promoting behaviors, suggesting that enhanced social support coincides with healthier behavioral patterns in this population. Social support may be connected to an individual's health and well-being by providing access to various physical resources that promote health (45). These mechanisms collectively show a relationship with frailty and thereby may coincide with the status of CHF and its related complications. Therefore, clinical management should prioritize the systematic assessment of a patient's social support, granting it the same importance as evaluations of physical function and nutritional status. Additionally, patients and their families should be encouraged and assisted in establishing or joining peer support groups, where shared experiences can enhance self-management confidence. Family members should also be guided in providing effective emotional support and practical care, thereby potentially being linked to better treatment adherence. Community resources should be leveraged to provide regular visits and organize social activities for patients living alone thereby enhancing their social connectedness and enabling more holistic health management.

This study found significant positive multiplicative and additive interactions between depressive symptoms and social support in relation to frailty, based on an analysis of patients with CHF from a Chinese tertiary hospital. To our knowledge, this is the first study to report both multiplicative and additive interactions between depressive symptoms and social support on frailty risk in a Chinese CHF population. The synergistic interaction can be explained by three interconnected pathways. First, from a psychosocial perspective, the stress-buffering model posits that social support protects against the adverse health effects of chronic stressors. In CHF patients, depressive symptoms act as a persistent stressor that activates the hypothalamic-pituitary-adrenal axis and sympathetic nervous system, leading to elevated cortisol and pro-inflammatory cytokines. High social support may attenuate this stress response by providing emotional comfort, practical assistance, and positive feedback, thereby reducing the physiological burden that promotes frailty. Second, behavioral pathways are involved. Depressive symptoms often reduce physical activity, impair self-care, and decrease treatment adherence—all factors linked to muscle wasting and functional decline. Low social support fails to counteract these behavioral changes, whereas high social support can encourage healthy behaviors and facilitate access to healthcare resources. Third, inflammatory mechanisms may underlie the interaction. Depression is associated with elevated levels of interleukin-6 and C-reactive protein, which contribute to sarcopenia and frailty. Social support has been shown to down-regulate inflammatory responses. Thus, the combination of depressive symptoms and low social support may result in a greater inflammatory burden than the sum of each alone, accelerating the development of frailty. These pathways are not mutually exclusive and likely reinforce each other. The finding that the interaction was strongest in older patients and those with NYHA class III further supports the clinical relevance of these mechanisms. Overall, the synergistic interaction reflects a vicious cycle where depressive symptoms and low social support jointly amplify biological, behavioral, and psychological risks, leading to a higher frailty burden. Consistent with prior studies, lower levels of social support may be correlated with depressive symptoms by undermining psychological well-being and decreasing participation in leisure physical activities (46), while loneliness resulting from low social support has also been linked to a higher likelihood of depressive symptoms, potentially mediated by neuroendocrine and immune pathways (47).

Subgroup analyses further revealed that the protective interaction of social support was particularly pronounced in older patients and those with NYHA class III. The observation that the interaction was strongest in older patients and those with NYHA class III is clinically plausible. Older patients often have fewer physiological reserves, making the buffering effect of social support more critical. Similarly, patients with moderate heart failure (NYHA III) may still have sufficient cognitive and physical capacity to benefit from social support, whereas those with advanced heart failure may already have reached a state of high frailty burden that is less modifiable by psychosocial factors. No significant interaction was observed in younger patients or in NYHA class II or IV (all *P* > 0.05). Notably, the interaction was significant in both LVEF < 50% and LVEF ≥ 50% subgroups, indicating that the modifying effect of social support is consistent across different levels of systolic function.

Consistent with prior studies (48–50), factors such as NYHA class, hypertension, and coronary heart disease were identified as significant correlates of frailty in patients with CHF. Gou et al. (51) found that higher NYHA class was linked to a higher likelihood of frailty in elderly patients with CHF. With worsening disease status, patients experience declining self-care, daily activity, and adaptive capacity, which coincides with severely limited exercise tolerance and depleted physiological reserve, thereby potentially showing a connection to muscle atrophy and functional decline that accompany frailty. Our findings also confirm that hypertension coincides with frailty, likely because suboptimal blood pressure control sustains increased cardiac afterload, which may be related to myocardial remodeling and functional decline, and when combined with frequent multimorbidity burden, may be linked to a higher frailty status (52). Furthermore, patients with coronary heart disease also exhibit a higher likelihood of frailty. Coronary atherosclerosis may be connected to frailty by causing chronic myocardial ischemia that correlates with impaired cardiac and physical function, while the systemic inflammatory state of the disease may also directly coincide with muscle metabolic dysfunction and the frailty syndrome (53). Therefore, clinicians should develop and implement individualized intervention strategies focused on frailty prevention and control within the comprehensive management of patients with CHF. Clinical management should integrate proactive frailty screening into the care of CHF patients with NYHA class II–IV. This involves developing individualized plans that combine nutrition, resistance training, and safety measures to address functional decline. Management of CHF patients with hypertension requires optimized control of both blood pressure and other comorbidities, with frailty prevention integrated into their long-term care objectives. For patients with CHF and comorbid coronary artery disease, management should extend beyond revascularization and pharmacotherapy to include monitoring of systemic inflammation and physical function. Early integration of exercise rehabilitation and nutritional intervention is recommended to address the key pathophysiological pathways linked to frailty. Implementing proactive multidimensional strategies for high-risk individuals may be correlated with a lower frailty incidence and improved heart failure prognosis.

This study has several innovative aspects. First, it is the first to simultaneously examine multiplicative and additive interactions between depressive symptoms and social support on frailty risk in a Chinese CHF population, providing a more comprehensive understanding of how these two psychosocial factors jointly relate to frailty. Second, the study is grounded in the biopsychosocial model, offering a theoretical framework that integrates biological, psychological, and social determinants of frailty. Third, the use of both interaction measures allows for a nuanced interpretation of effect modification and excess risk, which has important implications for risk stratification and intervention targeting. Based on these findings, routine screening for both depressive symptoms and social support should be integrated into standard CHF management to identify high-risk individuals, particularly those presenting with both depressive symptoms and low social support, who should be prioritized for comprehensive interventions that address both psychological well-being and social connectedness. Cardiac rehabilitation programs may benefit from incorporating psychosocial components such as cognitive behavioral therapy, peer support groups, and family involvement alongside traditional exercise and nutritional interventions, with multidisciplinary care teams collaborating to develop individualized care plans. For future research, longitudinal studies are needed to explore the temporal sequence of these relationships, intervention studies are warranted to evaluate the effectiveness of integrated psychosocial and physical rehabilitation programs in relation to frailty risk reduction, and multi-center studies with more diverse populations are needed to validate and extend our findings.

## Limitations

This study has several limitations. First, this was a single-center study in a specific region of China with a predominantly male sample (70.5%), which may limit the generalizability of our findings to other populations or settings. Second, the use of self-report questionnaires to assess depressive symptoms and social support may introduce recall and social desirability bias, potentially affecting the accuracy of the measurements. Third, the study sample was recruited from a single hospital and primarily comprised hospitalized patients, which may limit the representative of our findings for the broader population of patients with CHF in China. Additionally, the study sample had a predominance of male participants, further limiting the generalizability of our findings to female patients with CHF. Fourth, the cross-sectional nature of this study precludes causal inferences. While relationships were identified, the temporal sequence and directionality of relationships among depressive symptoms, social support, and frailty cannot be established. Future longitudinal studies are needed to elucidate the potential pathways linking these variables. Despite these limitations, our findings still carry significant clinical implications. Clinicians may consider integrating routine psychosocial screening using validated tools into standard assessments to identify patients with depressive symptoms and low social support. Such screening may be helpful in guiding emotional support, facilitating family involvement, and implementing individualized care plans that address both physical and psychosocial needs in this population.

## Conclusion

In conclusion, this study focused on patients with CHF, aiming to examine the relationships among depressive symptoms, social support, and frailty. Our findings indicate that both depressive symptoms and low social support are correlated with frailty in this population, and they demonstrate an additive interaction effect. Notably, the relationship of depressive symptoms with frailty was particularly pronounced. Furthermore, a significant multiplicative interaction suggests that higher social support coincides with a weaker relationship between depressive symptoms and frailty. These findings underscore the critical necessity of integrating psychosocial assessment into the routine management of CHF. Clinical practice should prioritize the early identification of patients experiencing both depressive symptoms and inadequate social support. Implementing multidimensional intervention strategies that synergistically address mental health and enhance social support networks is essential to address the detrimental biopsychosocial cycle and may be linked to a lower likelihood of frailty in this vulnerable population. Findings should be interpreted with caution and validated in more diverse populations.

## Data Availability

The raw data supporting the conclusions of this article will be made available by the authors, without undue reservation.
